# Age-Matching in Pediatric Fecal Matter Transplants

**DOI:** 10.3389/fped.2021.603423

**Published:** 2021-07-16

**Authors:** Abigale D. MacLellan, B. Brett Finlay, Silke Appel-Cresswell

**Affiliations:** ^1^Department of Integrated Sciences, University of British Columbia, Vancouver, BC, Canada; ^2^Michael Smith Laboratories, University of British Columbia, Vancouver, BC, Canada; ^3^Department of Microbiology and Immunology, University of British Columbia, Vancouver, BC, Canada; ^4^Department of Biochemistry and Molecular Biology, University of British Columbia, Vancouver, BC, Canada; ^5^Pacific Parkinson's Research Centre, Djavad Mowafaghian Centre for Brain Health, University of British Columbia, Vancouver, BC, Canada; ^6^Division of Neurology, Faculty of Medicine, University of British Columbia, Vancouver, BC, Canada

**Keywords:** FMT, donor, age-matching, disease, marker, pediatric, microbiome

## Introduction

Pediatric fecal matter transplant (FMT) clinical trials relying on “healthy” donor stool use screened adult donor stool, likely assuming safety and efficacy. However, we consider how adult donor stool could instead be posing risks of atypical development or transmission of early disease, which could be prevented with age-matching donors and recipients for FMT.

## Discussion

### Why Consider Age-Matching?

The gut microbiome has been identified as a potential therapeutic target to combat pediatric intestinal and extraintestinal diseases through FMT. FMT involves the transfer of “nondiseased” stool into the gastrointestinal (GI) tract of an individual with the disease in attempts to replenish the gut microbiota and ameliorate adverse GI symptoms. Currently, no standard methodology exists for clinical trials investigating FMT efficacy. Key elements such as donor selection processes are often based on drastically different criteria but usually involve adult stool donors even in pediatric FMT clinical trials. We consider how the usage of adult donors may be of concern when considering the long-term implications of FMT on pediatric health. The pediatric microbiome plays an important role in immune, GI, and neurological development and to introduce adult donor stool to an already dysbiotic microbiome could lead to atypical maturation. Furthermore, selection of nondiseased stool is based on current screening protocols. Screened and deemed “nondiseased” adult donor microbiota could be showing early disease markers for diseases that are not yet visible with current donor screening protocols, putting pediatric recipients at risk for disease development. Current pediatric guidelines have yet to consider these specific pediatric risks, which could be jeopardizing the long-term health and development of pediatric patients and influencing the future of pediatric therapeutics.

### Gut Microbiota and Pediatric Development

The microbiota is largely shaped in early childhood. Antibiotic usage in early childhood has shown to increase the risk of acquiring conditions such as inflammatory bowel disease (IBD), asthma, allergy, and eczema (1, 2). This risk likely (at least partially) involves the contribution of a dysbiotic microbiome, which could be hindering typical immunological, GI, and even neurological development (2–4). Children with IBD, asthma, eczema, and other allergic diseases have shown characteristic microbiota profiles, indicative that the gut microbiota is involved with these aspects of atypical development (1, 2). In addition to atypical neurological development, dysbiotic gut microbiota could act as a risk factor contributing to the development of neurological diseases (5).

Throughout childhood, adolescence, and adulthood, age is associated with drastic differences in composition and functional gene potential of the microbiome (6). Age greatly influences the gut microbial community diversity and structure, and when child and adult microbiota are compared, children's microbiota is most similar to other children as compared to adults (6).

In a pediatric FMT clinical trial, considerations for donor stool should include both microbial factors, such as similarity of microbial species between donor and recipient, as well as factors for host intestinal tolerance toward the donor microbes. Because the gut microbiota of young children, adolescents, and adults are all distinct, the necessary microbiota across development is crucial for typical neurological, immunological, and GI development (2–4, 6). If during this influential time of development, adult stool is introduced into a child, this period of neurological, immunological, and GI development could be impacted, which could lead to atypical maturation. However, considerations for donor selection currently focus on immediate safety and efficacy in FMT clinical trials but do not consider the aspects of long-term safety and efficacy.

### Current Considerations for FMT Donor Selection

In the pediatric population, active FMT clinical trials include treatment of intestinal colonization of extended-spectrum resistant Enterobacteriaceae (NCT02543866), IBD (NCT03399188), autism spectrum disorder (NCT03426826), ulcerative colitis (NCT03582969), Crohn's disease (NCT03378167), and refractory *Clostridioides difficile* infection (rCDI) (NCT02134392). In these pediatric trials, the age of the stool donor is listed as either an adult or is not mentioned in study record details.

Methodology within FMT clinical trials vary with donor selection procedures being a particular area of heterogeneity (7). Fecal donor selection can involve questionnaires and interviews to rule out antibiotic usage in the preceding months of stool donation, history of autoimmune or atopic illness, history of GI illnesses, and risk factors for multidrug resistant organisms, chronic pain syndromes, metabolic syndromes, malignancies, or some neurological disorders (7). Donor blood and stool can be evaluated using tests for known enteric pathogens, and tests for hepatitis A, B, C, human immunodeficiency virus (HIV), and syphilis (8). However, the definition of “safe” stool depends on the study, as a meta-analysis of 168 clinical trial FMT publications showed tests for both HIV and hepatitis C were recorded in 98% of publications, whereas general enteric pathogen screens, cytomegalovirus, and Epstein-Barr virus testing were recorded in 48, 31, and 28% of publications, respectively (7).

Screening efforts are established to not only ensure stool viability but robustly reduce the chance of transmitting infection or active medical comorbidities from donor to recipient (8). Screening procedures are constantly evolving based on results of FMT trials to include more elaborate testing for pathogens. The results of FMT trials stem from study design and reporting of methodology; however, many studies not only have heterogeneity in methodology but ambiguity as well. This ambiguity makes reproducible measures of FMT efficacy and safety more difficult to establish, even within treatment for the same disease.

Donor selection methodology is a particularly large area of ambiguity. For example, a systematic review of FMT clinical trials reported before 2017 determined that out of 85 FMT clinical trials, almost 50% of published studies did not report donor selection eligibility criteria in their publications (9). This reveals the reality of scarce reporting in donor selection making reproducibility and confident measures of trial efficacy almost impossible.

Trial method heterogeneity and uncertainty surrounding true efficacy further exacerbates the concerns for safety in FMT clinical trials. Safe stool is as safe as how it has been screened, and screening can only include testable pathogens already established to directly cause disease. However, the definition of what can cause disease is constantly evolving. For example, FMT has revealed the possibility of transmission of diseases previously assumed as nontransmissible. A patient developed issues with significant weight gain following an FMT from a healthy, but overweight, donor leading to FMT guidelines to suggest screening for donor body mass index (10). This possibility for transmission and disease manifestation was brought to researcher's attention based on the presentation of weight change in the patient occurring shortly after transplantation during an established follow-up period. This means the FMT follow-up period can be crucial to determine transmission possibilities and allow for the evolution of clinical FMT guidelines. However, current follow-up periods post-FMT are heterogeneous as well, meaning transmission of conditions with delayed manifestation might be missed. This is more problematic when considering pediatric FMT, when recipients have a lifelong potential for transmission and subsequent disease manifestation.

Current pediatric guidelines suggest donor selection should involve adults aged 18 and older based on medicolegal considerations (11). While adult donors are used to address immediate concerns for pediatric health, considerations for long-term safety and efficacy could be overlooked as transplanting an adult microbiota into a pediatric recipient could have long-term impacts on development and aging. Age-matching of donor and recipient for FMT begins to address microbiome-related donor concerns yet introducing pediatric stool donation requires additional thoughtful discussions surrounding consent and assent. In a letter written to the FDA outlining current consensus guidance on donor screening and stool testing for FMT, The Joint Society Consensus Recommendation suggests that “children could also potentially serve as donors as long as both parental consent and child assent are obtained” (12). This letter proposes that children can act as FMT donors; however, before clinical trials can occur with pediatric donors, a further examination of medicolegal considerations surrounding child assent and caregiver consent specific for stool donation is crucial.

### Could Adult Donor Stool Transmit Disease Markers to Pediatric Recipients?

Early life disruptions to the microbiota can already increase an individual's susceptibility to a variety of immune, metabolic, neurological, and psychiatric diseases (13). This phenomenon could be exacerbated by an adult microbiota that could potentially be transmitting early disease markers.

Early disease markers for a variety of gut microbiome associated diseases have not yet been identified. These early disease markers could be characteristic changes occurring in the gut of an adult donor, years before diagnosis of disease, and invisible with current stool-screening guidelines. It is established that bidirectional communication between the brain and GI tract has been implicated in a variety of neurological diseases. Transmission of early disease markers from a variety of diseases could occur; here, we will illustrate the issue using Parkinson's disease (PD) as an example. PD has characteristic changes in the GI tract including constipation and gut dysbiosis with increased inflammation and reduced short-chain fatty acid production. Adults with REM-sleep behavior disorder, a prodromal stage of PD, already have the same pattern of dysbiosis (14). Constipation and other GI comorbidities can present up to 20 years before PD motor symptoms arise (15). Furthermore, the gut microbiome plays a pivotal role in the manifestation of PD-like symptoms in a PD mouse model (16). This model showed that germ-free animals developed typical PD pathology, GI, and central nervous system symptoms only upon introduction of the gut microbiome (16). Crucially, FMTs from human donors with PD led to worse motor symptoms in the receiving mice compared to mice receiving FMTs from healthy human donors (16).

To contextualize early disease marker transmission using specific evidence from pediatric FMT, a study of the gut microbiome of pediatric recipients following FMT with “healthy” adult donor stool showed the potential for acquisition and persistence of antimicrobial genes from the donor (17). Although the genes acquired were tetracycline antimicrobial resistance genes, which are commonly found in adults and children and not of particular concern, the authors caution that acquisition of clinically significant antimicrobial resistance genes are possible and could be especially dangerous if pediatric FMT recipients are immunocompromised, as many are (17). Furthermore, growing evidence suggests a role for microbes as early disease markers in colorectal cancer (18). A study of pediatric FMT recipients for rCDI showed these procarcinogenic bacteria originating from adult donors were transmitted and reconstituted in the gut microbiome of recipients up to 6 months following their FMT (18).

We are concerned that adult donors beginning to generate characteristic microbiome profiles of adult-onset disease could be acting as FMT donors for pediatric FMT trials, potentially generating long-term implications for the recipient child with their GI tract being introduced to characteristic pathogenic changes in childhood. Matching pediatric FMT recipients with pediatric donors could largely reduce the possibility of adult-onset neurodegenerative disease marker transmission and allow for less interruption of age-specific maturation of the gut microbiota.

### Is Age-Matching a Perfect Match?

With past efforts focused on the promotion of immediate safety for pediatric FMT recipients, the field is ready for conversations surrounding long-term safety and healthy development in pediatric FMT recipients. Optimal donor selection should be a focus of these conversations, with considerations of matching donors and recipients by age being prioritized. Age-matching could reduce the exacerbation of the child's atypical immune, GI, metabolic, and neurological development to allow for parallel interplay of microbiota development with typical physiological development.

The idea of age-matching is not without an introduction of its own novel risks, such as possible transmission of childhood-onset diseases including asthma or allergy to pediatric recipients. However, we argue this phenomenon could still occur with the introduction of adult donor microbiota because it could be affecting typical immunological development. Furthermore, screening for risk factors for allergy and asthma, such as antibiotic usage, mode of delivery, and formula feeding in pediatric donors, could aid to protect against this occurrence.

A joint-position paper from the North American Society for Pediatric Gastroenterology, Hepatology, and Nutrition and the European Society for Pediatric Gastroenterology, Hepatology, and Nutrition similarly argues that further research into age-matching is warranted due to concerns of long-term efficacy and safety with adult donors (11). Age-matching is influential when considering development and maturation, and also includes a further safety advantage as children acting as donors would tend to have less environmental exposure and would be less likely to carry transmittable infections from travel, illicit drug use, or sexual activity (8).

Currently pediatric FMTs are changing children's lives, treating persistent, and sometimes otherwise incurable, diseases, demonstrating the power of the approach. FMT trials explore increasingly diverse applications for pediatric intestinal and extraintestinal diseases and long-term clinical considerations for health and development will be increasingly important. Given the increasingly recognized role of the gut microbiome in adult diseases including neurodegenerative disorders, we believe choosing age-matched donors for pediatric FMTs will likely be the best safe-guard to reduce potential future complications.

## Footnotes

At time of submission all clinical trials statuses mentioned in the manuscript are listed as: NCT02543866 (recruiting), NCT03399188 (recruiting), NCT03426826 (recruiting), NCT03582969 (recruiting), NCT03378167 (recruiting), and NCT02134392 (recruiting).

Current trial statuses can be accessed at https://clinicaltrials.gov/.

## Author Contributions

AM conceived the opinion and drafted manuscript. BF and SA-C contributed expertise to review and critique final manuscript. All authors contributed to the article and approved the submitted version.

## Conflict of Interest

The authors declare that the research was conducted in the absence of any commercial or financial relationships that could be construed as a potential conflict of interest.
